# Evidence for Publicly Reported Quality Indicators in Residential Long-Term Care: A Systematic Review

**DOI:** 10.1093/geroni/igaa057.586

**Published:** 2020-12-16

**Authors:** Franziska Zúñiga, Magdalena Osinska, Franziska Zuniga

**Affiliations:** 1 Basel University, Basel, Basel-Stadt, Switzerland; 2 University of Basel, Basel, Switzerland

## Abstract

Quality indicators (QIs) are used internationally to measure, compare and improve quality in residential long-term care. Public reporting of such indicators allows transparency and motivates local quality improvement initiatives. However, little is known about the quality of QIs. In a systematic literature review, we assessed which countries publicly report health-related QIs, whether stakeholders were involved in their development and the evidence concerning their validity and reliability. Most information was found in grey literature, with nine countries (USA, Canada, Australia, New Zealand and five countries in Europe) publicly reporting a total of 66 QIs in areas like mobility, falls, pressure ulcers, continence, pain, weight loss, and physical restraint. While USA, Canada and New Zealand work with QIs from the Resident Assessment Instrument – Minimal Data Set (RAI-MDS), the other countries developed their own QIs. All countries involved stakeholders in some phase of the QI development. However, we only found reports from Canada and Australia on both, the criteria judged (e.g. relevance, influenceability), and the results of structured stakeholder surveys. Interrater reliability was measured for some RAI QIs and for those used in Germany, showing overall good Kappa values (>0.6) except for QIs concerning mobility, falls and urinary tract infection. Validity measures were only found for RAI QIs and were mostly moderate. Although a number of QIs are publicly reported and used for comparison and policy decisions, available evidence is still limited. We need broader and accessible evidence for a responsible use of QIs in public reporting.

